# Validation of a deep-learning based thrombus classifier on digital subtraction angiography using a large-scale dataset

**DOI:** 10.1007/s00234-026-04046-0

**Published:** 2026-06-04

**Authors:** Johannes Rosskopf, Aliye Yazilitas, Sarah Elfeel, Timo Baumgärtner, Alfred Michael Franz, Robert Mueller, Michael Ertl, Bernd Schmitz, Katharina Althaus

**Affiliations:** 1 Section of Neuroradiology, Bezirkskrankenhaus Guenzburg, Guenzburg, Germany; 2 Department of Radiology, University Hospital Ulm, Ulm, Germany; 3 Department of Computer Science, University of Applied Sciences Ulm (THU), Ulm, Germany; 4 Institute for AI Safety and Security, German Aerospace Center (DLR), Ulm, Germany; 5 Division of Intelligent Medical Systems, German Cancer Research Center (DKFZ), Heidelberg, Germany; 6 Department of Neurology, Bezirkskrankenhaus Guenzburg, Guenzburg, Germany; 7 Department of Neurology, Christophsbad Medical Center, Goeppingen, Germany

**Keywords:** Digital subtraction angiography, Deep-learning classifier, Thrombus, Stroke, Real-world data

## Abstract

**Purpose:**

Digital subtraction angiography (DSA) interpretation is observer dependent. This study evaluated the diagnostic performance of an existing deep-learning (DL) based thrombus classifier prior to clinical application. The intended use of the model is as a clinical decision-support tool to assist neuroradiological assessment during mechanical recanalization.

**Methods:**

This retrospective study included an in-house dataset of DSA image series from endovascular recanalization procedures for anterior circulation occlusions collected over two years. For each case, two DSA runs were selected: one before and one after recanalization. The artificial intelligence system was applied to classify thrombus presence. Diagnostic performance was assessed using sensitivity, specificity, and false-positive rate.

**Results:**

A total of 1,236 DSA series from 309 patients were analyzed, yielding 618 paired biplane acquisitions. The DL classifier achieved an overall sensitivity of 71.7% (95% CI 67.3–75.8%), with the highest sensitivity for proximal vessel occlusions (M1/M2 segments: 87.6%; 95% CI 83.6–90.9%) and substantially lower sensitivity for distal occlusions (M3/M4 segments: 23.1%; 95% CI 14.9–33.1%) as well as for occlusions of the anterior cerebral artery (27.3%; 95% CI 10.7–50.2%). Overall specificity for thrombus detection was 89.8% (149/166) (95% CI: 84.1–93.9%), corresponding to 17 false-positive classifications.

**Conclusion:**

The developed DL classifier on DSA series confirmed on a large-scale dataset its very high sensitivity to proximal vessel occlusions with a sensitivity of 87.6%. Sensitivity for distal vessel occlusions was very low. Training the system for these lesion types will be the next step prior to clinical application.

## Introduction

Digital subtraction angiography (DSA) is a catheter-based real-time imaging technique routinely used during endovascular treatment of acute ischemic stroke [1, 2]. Endovascular recanalization has been shown to significantly improve clinical outcome compared with medical treatment alone [3]. Accurate and timely identification of intracranial vessel occlusions on DSA is therefore essential for procedural guidance and therapeutic decision-making. However, visual detection of vascular occlusions during the intervention may be challenging, particularly under time pressure and in complex vascular anatomies. Overlapping vessels within the imaging plane, severe vascular tortuosity or multiple occlusions may further impede reliable visual assessment [4–7]. The interobserver variability of DSA interpretation is known to be high [4, 8–10]. Therefore, automating DSA image assessment in acute ischemic stroke, aimed at reducing subjectivity and improving standardization, has recently been recommended by the STAIR (Stroke Treatment Academic Industry Roundtable) XII committee [11].

To date, automatic TICI (Thrombolysis In Cerebral Infarction) scoring models have primarily been developed to improve comparability of treatment success between clinical trials [4, 6, 12, 13]. Deep-learning (DL) based methods have been proposed to identify vessel occlusions on DSA to assist neuro-interventionalists [7, 14–17]. Although the current literature about artificial intelligence in interventional radiology remains at an early stage [5], substantial progress has been achieved over recent years. DL models have demonstrated improved performance in localizing intracranial vessel occlusions [7, 15]. In addition, advances in computational pipeline optimization have enabled near-real-time decision support during endovascular treatment with processing times of less than 5 s [14, 18, 19].

Despite these advances, the translation of DL models into clinical routine remains challenging [20]. Most DL models are developed and evaluated in a study setting using uniform datasets, which may restrict generalizability to real-world patient populations [7, 14, 15]. Consequently, model performance can vary when applied to heterogeneous clinical environments. Large-scale validation on unselected, previously unseen data is therefore considered essential to reliably assess robustness and clinical applicability [5, 20, 21].

The present study aimed to evaluate the diagnostic performance of an open-source DL based thrombus presence–absence classifier on a large-scale real-world clinical DSA dataset. The classifier was designed as a clinical decision-support tool to assist neuroradiological assessment during mechanical recanalization [14]. The tool has been previously trained on proximal vessel occlusions of the middle cerebral artery (M1 and M2 segments) and published open-source by our group [14, 19, 22]. The primary objective was to assess whether the sensitivity for thrombus detection in proximal vessel occlusions could be confirmed on an unseen dataset, and to determine the classification performance for more distally located, previously untrained occlusions as well as for occlusions at other locations within the anterior circulation. The results are intended to be considered hypothesis-generating and to provide guidance prior to translation of the system into routine clinical practice.

## Materials and methods

### Study design and patient selection

All patients who underwent endovascular treatment for acute intracranial vessel occlusion in a stroke setting were retrospectively identified from the institutional database over a two-year period. The current validation cohort is strictly independent of the training cohort, with no overlapping cases due to completely separated time intervals of data acquisition. However, both datasets originate from the same institution, scanner, and clinical workflow, which may result in distributional similarity. The training dataset previously included only M1/M2 occlusions and has now been expanded to anterior circulation occlusions.

For each case, two whole-brain DSA series were analyzed: one acquired before recanalization and one after completion of the procedure. Paired biplane acquisitions (posteroanterior and lateral views) were used as input for the pre-trained model to perform thrombus classification. Intraprocedural acquisitions with smaller fields of view were not included, as the model was not trained on such data and their inclusion would have introduced a significant confounding factor.

Inclusion criteria comprised acute occlusions of the middle cerebral artery at proximal (M1, M2) and distal segments (M3, M4), as well as occlusions of the anterior cerebral artery. Occlusion location was assessed by one board-certified neuroradiologists, with more than 5 years of experience. In cases of multiple vessel occlusions, classification was based on the most proximal occlusion.

Exclusion criteria included tandem occlusions of the proximal carotid artery and intracranial vessels, as well as posterior circulation occlusions, including the basilar and posterior cerebral arteries. Datasets were excluded if both posteroanterior and lateral DSA series were not available. The classifier is intended to assist interventionalist. Therefore, its primary focus is not on carotid-T occlusions, which are typically readily identifiable and do not require additional support from the classifier. Procedures acquired on the institutional monoplane angiography system were excluded. Only examinations performed on the biplanar Artis Icono system (Siemens Healthineers) were included. Cases with spontaneous recanalization and no angiographic evidence of vessel occlusion on the initial DSA run were also excluded. However, the degree of recanalization (e.g., TICI 0–3) did not influence study inclusion. All procedures were performed under general anesthesia, and no motion artifacts were present in the analyzed DSA series. Due to the retrospective study design, the requirement for written informed consent was waived. The study was approved by the Ethics Committee of Ulm University, Germany (No. 306/20) and was conducted in accordance with the 1964 Helsinki Declaration and its later amendments.

## Deep-learning classifier

The DL classifier was in-house developed and has been previously published open-source [14, 19, 22]. The system was designed to automatically classify DSA sequences as thrombus-free or non–thrombus-free based on occlusion-related perfusion abnormalities. DSA image series were treated as variable-length temporal sequences and analyzed using a combined convolutional and recurrent neural network architecture as described in the following.

**Fig. 1:**
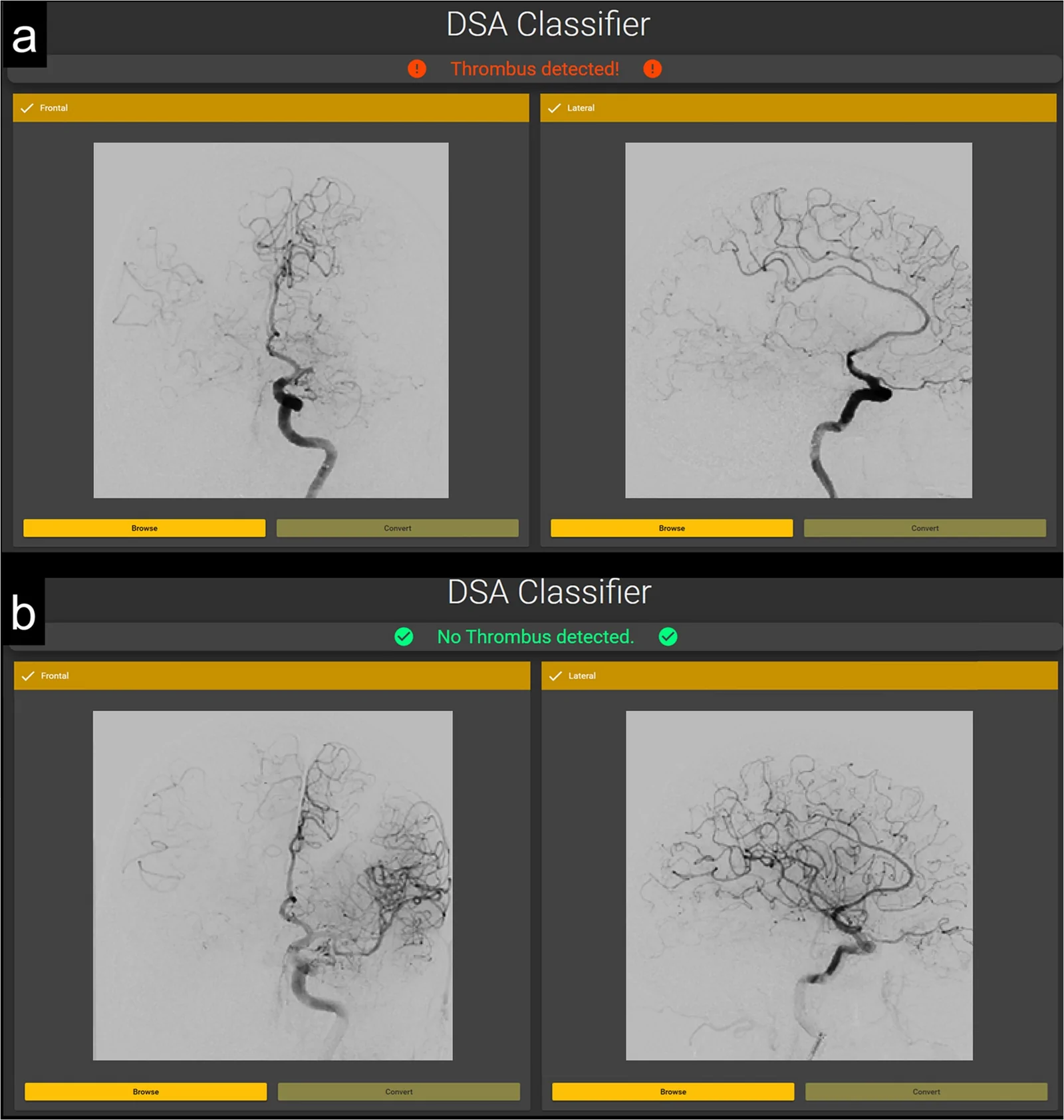
Graphical user interface of the deep-learning based thrombus classifier showing classification results for paired biplane digital subtraction angiography images. Example of correct thrombus detection in an M1 segment occlusion of the middle cerebral artery (**A**, red text) and correct classification of complete recanalization after endovascular treatment (**B**, green text)

Image preprocessing of the system [14] includes intensity normalization and resizing to a uniform spatial resolution. Spatial feature extraction is performed using convolutional neural network backbones pretrained on ImageNet. Temporal information reflecting contrast-agent propagation is modeled using recurrent neural networks. To implement the architecture, different ResNet18 and EfficientNet-based backbones were tested in combination with gated recurrent units (GRU) and long short-term memory (LSTM) [23] networks [14]. Separate models are trained for posterior–anterior and lateral angiographic projections to account for view-specific perfusion characteristics. For model inference, paired biplane acquisitions are analyzed jointly, and predictions from all networks are equally weighted to generate the final classification output [24, 25]. Based on the results of the different network variants [14], EfficentNetV2 combined with GRU was defined as the final architecture in a follow-up study [22]. It was used to train a model that is published open-source, including a classification application [22], and has been tested for live classification in a clinical setting [19]. Model training was performed using 1,068 DSA sequences from 260 patients corresponding to 534 paired posteroanterior and lateral acquisitions [14, 22]. Based on input posteroanterior and lateral DSA sequences, the network computes a probabilistic binary output indicating the presence or absence of thrombus-related perfusion abnormalities (Fig. 1). Classification is performed applying a default threshold of 0.57 on the network voting between 0 and 1. The default threshold was found by optimizing the rates of false positives and true positives in a previous study [14].

### Statistical analysis

The diagnostic performance of the DL classifier was assessed for the entire study cohort. Performance metrics included sensitivity, specificity, and false-positive rate, with corresponding confidence intervals reported. Confidence intervals were calculated using standard binomial methods. Diagnostic accuracy was additionally evaluated in predefined subgroups according to vessel occlusion location (M1, M2, M3, M4, ACA).

## Results

### Study population

A large-scale digital subtraction angiography (DSA) dataset comprising 1,236 DSA sequences was analyzed in this validation study. These data were derived from 309 patients with anterior circulation vessel occlusions and resulted in 618 paired diagnostic classification decisions. Among these, 452 cases were thrombus-positive and 166 thrombus-negative. The cohort included 165 occlusions of the M1 segment and 174 of the M2 segment, as well as more distal occlusions involving the M3 (*n* = 20) and M4 (*n* = 71) branches. In addition, 22 occlusions were located within the anterior cerebral artery, including the A1 (*n* = 2) and more distal segments (*n* = 20) (Table 1B).

**Table 1:** Diagnostic performance of the DL classifier

**A**			
	**DL: thrombus**		**DL: no thrombus**
**HR: thrombus**	*324*		128
**HR: no thrombus**	*17*		149
**B**			
**Localization** ^**1**^	***n*** ^**2**^	**Sensitivity (95%CI)** ^**3**^	
**M1**	165	97.0% (93.2–98.9%)	
**M2**	174	78.7% (71.9–84.5%)	
**M3**	20	30.0% (11.9–54.3%)	
**M4**	71	21.1% (12.3–32.4%)	
**A1**	2	50.0% (1.3–98.7%)	
**A2** ^4^	20	25.0% (8.7–49.1%)	

**(A)** Confusion matrix of the whole study cohort comparing human ratings (HR) with model prediction (DL) and **(B)** sensitivities of the deep-learning (DL) classifier for each occlusion location.

^1^anatomical localization of the vessel occlusions within the middle cerebral artery (M1-M4 segments) or the anterior cerebral artery.

^2^*n* = numbers.

^3^sensitivity to vessel occlusion with 95%CI (confidence interval).

^4^including A2 and more distal segments (A3/A4/A4)

### Diagnostic performance of the DL classifier

#### a) overall performance

Across the entire large-scale dataset, the DL classifier achieved an overall sensitivity of 71.7% (95% CI, 67.3%–75.8%), correctly identifying 324 of 452 thrombus-positive DSA series. Consequently, 128 thrombus-positive cases were misclassified as thrombus-negative, corresponding to a false-negative rate of 28.3%. For thrombus-negative DSA series, the classifier reached a specificity of 89.8% (95% CI, 84.1%–93.9%), with 149 of 166 no-thrombus cases correctly classified. This resulted in 17 false-positive classifications, corresponding to a false-positive rate of 10.2% (Table 1A). No consistent pattern could be identified among false-positive cases; notably, the model also classified cases with complete reperfusion as thrombus-positive.

Overall, these results indicate higher accuracy for exclusion of thrombus than for thrombus detection. However, these global performance metrics conceal relevant variations related to anatomical location, which are explored in more detail below.

#### b) localization specific performance

Classifier performance varied substantially according to occlusion location (Table 1B). Sensitivity was highest for proximal middle cerebral artery occlusions, with 97.0% (95% CI, 92.9%–99.2%) for M1 segments and 78.7% (95% CI, 71.9%–84.4%) for M2 segments. When combined, proximal MCA occlusions (M1/M2) yielded an overall sensitivity of 87.6% (95% CI, 83.6%–90.9%).

In contrast, diagnostic performance decreased markedly for more distal vessel occlusions. Sensitivity was 30.0% (95% CI, 11.9%–54.3%) for M3 and 21.1% (95% CI, 12.6%–32.6%) for M4 segment occlusions, resulting in a combined sensitivity of 23.1% (95% CI, 14.9%–33.1%) for distal MCA occlusions (M3/M4). This corresponds to a reduction in sensitivity of 64.5% points compared with proximal MCA segments.

Occlusions of the anterior cerebral artery were uncommon and showed similarly limited detection performance, with a combined sensitivity of 27.3% (95% CI, 10.7%–50.2%) for A1 and more distal segments.

To assess potential bias introduced by the structured pairing of positive and negative cases, sensitivities for each vessel occlusion location were calculated based on pre-recanalization runs only and remained within a comparable range (M1: 97.5%; M2: 83.1%; M3: 50.0%; M4: n.a.; A1: 50.0%; A2: 33.3%).

## Discussion

In this large-scale validation study, a DL classifier demonstrated robust diagnostic performance for proximal anterior circulation vessel occlusions. Sensitivity was high for proximal middle cerebral artery occlusions (M1/M2, 87.6%) but declined markedly for distal (M3/M4, 23.1%) and anterior cerebral artery occlusions (27.3%). As expected, robust performance is demonstrated for proximal targets on which the network was trained, but not for distal or anatomically unrepresented thrombi.

The current results are consistent with prior evaluations of the same DL classifier conducted on the in-house test dataset during model development [14]. Mittmann et al. previously reported a sensitivity of 86.1% and a specificity of 86.7% for M1/M2 segment occlusions [14], very closely corresponding to the current observed sensitivity of 87.6% and specificity of 89.8% for proximal vessel occlusions. This component of stress testing served as an internal validation using a previously unseen large-scale DSA dataset and confirmed model robustness beyond the original training cohort. However, this approach does not represent true external validation and therefore primarily reflects performance within a similarly distributed data environment. Overfitting remains a relevant concern for AI systems in radiology [26]. Specifically, it cannot be fully assessed to what extent image noise characteristics, overall image appearance, reconstruction algorithms, and angiography-specific features were implicitly learned during training, potentially limiting model performance to data acquired on the institution’s biplanar angiography system. Of note, underspecification, as the second major barrier to generalizability [26], is generally considered less critical in the acute stroke setting, as large-vessel occlusion constitutes a well-defined pathophysiological entity with a direct causal link between thrombus formation and vascular occlusion.

When compared with previously published DSA-only studies, the diagnostic performance of the present DL classifier falls within a comparable range for the detection of proximal vessel occlusions [7, 15, 16]. Warman et al. developed a DL approach trained on a DSA cohort of 188 patients with M1 and proximal M2 segment occlusions, achieving a sensitivity of 76% and a specificity of 95% [15]. In contrast, Khankari et al. reported higher diagnostic performance for their automated DL detection system, with a sensitivity of 93% and a specificity of 91%, most likely attributable to the predominance of occlusions of the internal carotid artery and M1 segment within their study population [7].

Detection performance was markedly lower for distal vessel occlusions (M3/M4 segments, 23.1%) and anterior cerebral artery occlusions (27.3%). The most plausible explanation is that the DL classifier was not trained on these occlusion locations. Consequently, generalizability of the present DL classifier to all occlusions of the anterior circulation cannot be assumed. Notably, even previous studies on automated TICI scoring systems have not evaluated distal vessel occlusions. Nielsen et al. restricted their analysis to proximal occlusions [6]. Van der Sluijs et al. evaluated automated eTICI scoring in patients with large-vessel occlusions up to the M2 segment [4]. The authors reported a significantly weaker association between automated TICI (autoTICI) and expert-derived eTICI scores for M2 occlusions compared with more proximal occlusion locations [4].

The present validation process revealed several areas for further improvement of the DL classifier. In particular, the large-scale dataset could enable training for distal vessel occlusions, which are more difficult to assess on DSA due to smaller vessel calibers, reduced contrast opacification and vessel overlap [20]. A system optimized for distal occlusions could therefore provide substantial clinical value. Our preliminary optimization experiments suggest that a stronger weighting of the lateral view may improve detection, as distal occlusions are often difficult to detect on the posteroanterior view; however, this requires further validation. Furthermore, while real-time analysis with a dedicated interface to the angiography system in the control room has already been implemented, with results available in under five seconds [19], integration of the DL graphical user interface into the display monitor at the intervention table is currently lacking and represents an important step toward full clinical workflow integration. According to the manufacturer, this is technically feasible, while ethical aspects are still under discussion. In addition, automated thrombus localization would be desirable. However, high interobserver variability in thrombus position annotation currently limits objective performance assessment [14]. Previous studies have similarly emphasized still unresolved challenges regarding annotation variability and suitable evaluation metrics for thrombus detection tasks [7, 15].

This study has several limitations. First, although the dataset represents the largest cohort to date investigating DL thrombus classification on DSA, it remains relatively small by DL standards. Nevertheless, the primary objective of this study, i.e. to evaluate the performance of a previously developed DL model when applied to real-world clinical datasets, could be adequately addressed. Second, validation was restricted to an internal dataset. Consequently, no conclusions can be drawn regarding model performance across external populations, different ethnic groups, angiography systems from other vendors, or varying procedural techniques among neurointerventionalists. The absence of a second neuroradiologist precluded assessment of interrater agreement. In the previously published paper on the DL classifier [14], the DSA sequences were annotated by two experienced neuroradiologists. As the results with respect to M1/M2 detection can be confirmed with the current dataset, no major issues related to inter-reader variability are expected. Furthermore, vascular pathologies such as high-grade stenoses or non-occlusive lesions were not evaluated, despite their clinical relevance during endovascular procedures. The performance of the DL classifier in these cases remains unknown. Additionally, the ability of the model to differentiate between thrombotic occlusions and other intraluminal or flow-limiting abnormalities therefore remains to be investigated in future studies.

## Conclusion

In this large-scale validation study, a DL classifier demonstrated robust diagnostic performance for proximal middle cerebral artery occlusions (M1/M2 segments), with a sensitivity of 87.6%, while performance declined substantially for distal and anterior cerebral artery occlusions, with sensitivities below 30%. Diagnostic accuracy for proximal occlusions was consistent with prior in-house testing. These findings highlight the anatomical dependency of current DSA-based AI systems. Future development should focus on extending model training to distal vessel occlusions and on validating the model using external data from other clinics before routine clinical implementation.

## Data Availability

The data that support the findings of this study are not openly available due to reasons of sensitivity and are available from the corresponding author upon reasonable request. Data are located in controlled access data storage at University of Ulm.

## References

[1] Psychogios M, Brehm A, Ribo M, Rizzo F, Strbian D, Räty S et al (2025) Endovascular Treatment for Stroke Due to Occlusion of Medium or Distal Vessels. N Engl J Med 392(14):1374–1384 10.1056/NEJMoa2408954 39908430

[2] Costalat V, Jovin TG, Albucher JF, Cognard C, Henon H, Nouri N et al (2024) Trial of Thrombectomy for Stroke with a Large Infarct of Unrestricted Size. N Engl J Med 390(18):1677–1689 10.1056/NEJMoa2314063 38718358

[3] Goyal M, Menon BK, van Zwam WH, Dippel DWJ, Mitchell PJ, Demchuk AM et al (2016) Endovascular thrombectomy after large-vessel ischaemic stroke: a meta-analysis of individual patient data from five randomised trials. Lancet 387(10029):1723–1731 10.1016/S0140-6736(16)00163-X 26898852

[4] van der Sluijs PM, Su R, Cornelissen S, van Es ACGM, Lycklama A, Nijeholt GJ, van Doormaal PJ et al (2025) Assessment of automated TICI scoring during endovascular treatment in patients with an ischemic stroke. J Neurointerv Surg 17(8):830–836 10.1136/jnis-2024-021892 39019506

[5] Glielmo P, Fusco S, Gitto S, Zantonelli G, Albano D, Messina C et al (2024) Artificial intelligence in interventional radiology: state of the art. Eur Radiol Exp 8(1):62 10.1186/s41747-024-00452-2 PMC11063019 38693468

[6] Nielsen M, Waldmann M, Frölich AM, Flottmann F, Hristova E, Bendszus M et al (2021) Deep Learning-Based Automated Thrombolysis in Cerebral Infarction Scoring: A Timely Proof-of-Principle Study. Stroke 52(11):3497–3504 10.1161/STROKEAHA.120.033807 34496622

[7] Khankari J, Yu Y, Ouyang J, Hussein R, Do HM, Heit JJ et al (2023) Automated detection of arterial landmarks and vascular occlusions in patients with acute stroke receiving digital subtraction angiography using deep learning. J Neurointerv Surg 15(6):521–525 10.1136/neurintsurg-2021-018638 35483913

[8] Ibrahim MK, Shehata MA, Ghozy S, Bilgin C, Jabal MS, Heiferman DM et al (2023) Operator assessment versus core laboratory adjudication of recanalization following endovascular treatment of acute ischemic stroke: a systematic review and meta-analysis. J Neurointerv Surg. ;15(2):133 LP – 138 10.1136/jnis-2022-019266 36163347

[9] Bar M, Mikulik R, Jonszta T, Krajina A, Roubec M, Skoloudik D et al (2012) Diagnosis of recanalization of the intracranial artery has poor inter-rater reliability. AJNR Am J Neuroradiol 33(5):972–974 10.3174/ajnr.A2896 PMC7968815 22241381

[10] Heiferman DM, Pecoraro NC, Wozniak AW, Ebersole KC, Jimenez LM, Reynolds MR et al (2020) Reliability of the Modified TICI Score among Endovascular Neurosurgeons. AJNR Am J Neuroradiol 41(8):1441–1446 10.3174/ajnr.A6696 PMC7658888 32719092

[11] Prasetya H, Ramos LA, Epema T, Treurniet KM, Emmer BJ, van den Wijngaard IR et al (2021) qTICI: Quantitative assessment of brain tissue reperfusion on digital subtraction angiograms of acute ischemic stroke patients. Int J stroke Off J Int Stroke Soc 16(2):207–216 10.1177/1747493020909632 PMC7859588 32098584

[12] Nielsen M, Waldmann M, Sentker T, Frölich A, Fiehler J, Werner R (2020) Time Matters: Handling Spatio-Temporal Perfusion Information for Automated TICI Scoring. In: Martel AL, Abolmaesumi P, Stoyanov D, Mateus D, Zuluaga MA, Zhou SK et al (eds) Medical Image Computing and Computer Assisted Intervention -- MICCAI 2020. Springer International Publishing, Cham, pp 86–96

[13] Su R, Cornelissen SAP, van der Sluijs M, van Es ACGM, van Zwam WH, Dippel DWJ et al (2021) autoTICI: Automatic Brain Tissue Reperfusion Scoring on 2D DSA Images of Acute Ischemic Stroke Patients. IEEE Trans Med Imaging 40(9):2380–2391 10.1109/TMI.2021.3077113 33939611

[14] Mittmann BJ, Braun M, Runck F, Schmitz B, Tran TN, Yamlahi A et al (2022) Deep learning-based classification of DSA image sequences of patients with acute ischemic stroke. Int J Comput Assist Radiol Surg 17(9):1633–1641 10.1007/s11548-022-02654-8 PMC9463240 35604489

[15] Warman R, Warman PI, Warman A, Bueso T, Ota R, Windisch T et al (2024) A deep learning method to identify and localize large-vessel occlusions from cerebral digital subtraction angiography. J neuroimaging Off J Am Soc Neuroimaging 34(3):366–375 10.1111/jon.13193 38506407

[16] Kelly B, Martinez M, Do H, Hayden J, Huang Y, Yedavalli V et al (2023) DEEP MOVEMENT: Deep learning of movie files for management of endovascular thrombectomy. Eur Radiol 33(8):5728–5739 10.1007/s00330-023-09478-3 PMC10326097 36847835

[17] Schuldhaus D, Spiegel M, Redel T, Polyanskaya M, Struffert T, Hornegger J et al (2011) Classification-based summation of cerebral digital subtraction angiography series for image post-processing algorithms. Phys Med Biol 56(6):1791–1802 10.1088/0031-9155/56/6/017 21346277

[18] Windecker S, Kolh P, Alfonso F, Collet J-PP, Cremer J, Falk V et al (2014) 2014 ESC/EACTS Guidelines on myocardial revascularization: The Task Force on Myocardial Revascularization of the European Society of Cardiology (ESC) and the European Association for Cardio-Thoracic Surgery (EACTS)Developed with the special contribution o. Eur Heart J 35(37):2541–2619 10.1093/eurheartj/ehu278 25173339

[19] Komposch J, Malzacher T, Baumgärtner T, Braun M, Roßkopf J, Franz AM et al (2024) A setup for live AI support in interventional radiology. Curr Dir Biomed Eng 10(4):385–388

[20] Kunapinun A, Suthakorn J, Siravaman D, Songsaeng D Toward AI-Powered Neurovascular Intervention: 2026;(April) 10.1161/STROKEAHA.125.053121 41376583

[21] Kono K (2025) Artificial Intelligence in Neuroendovascular Procedures. J neuroendovascular Ther 19(1):2024–0107 10.5797/jnet.ra.2024-0107 PMC11873741 40034100

[22] Baumgärtner T, Mittmann BJ, Malzacher T, Roßkopf J (2023) Towards clinical translation of deep-learning based classification of DSA image sequences for stroke treatment. Inf aktuell Proc Ger Work Med Image Comput. pp 95–101

[23] Hochreiter S, Schmidhuber J (1997) Long Short-Term Memory. Neural Comput 9(8):1735–1780 10.1162/neco.1997.9.8.1735 9377276

[24] Narang S, Diamos G, Elsen E, Micikevicius P, Alben J, Garcia D et al (2017) Mixed Precision Training. 6th Int Conf Learn Represent ICLR 2018 - Conf Track Proc

[25] Loshchilov I, Hutter F (2017) Decoupled Weight Decay Regularization. 7th Int Conf Learn Represent ICLR 2019

[26] Eche T, Schwartz LH, Mokrane F-Z, Dercle L (2021) Toward Generalizability in the Deployment of Artificial Intelligence in Radiology: Role of Computation Stress Testing to Overcome Underspecification. Radiol Artif Intell 3(6):e210097 10.1148/ryai.2021210097 PMC8637230 34870222

